# Crystal structure of poly[[hexa­aqua­tris­(μ-3,6-di­oxo­cyclo­hexa-1,4-diene-1,4-diolato)dierbium(III)] octa­deca­hydrate]

**DOI:** 10.1107/S2056989018017516

**Published:** 2019-01-01

**Authors:** Nutcha Ponjan, Kenika Kodchasanthong, Suwadee Jiajaroen, Kittipong Chainok

**Affiliations:** a Materials and Textile Technology, Faculty of Science and Technology, Thammasat University, Khlong Luang, Pathum Thani 12121, Thailand; b Division of Chemistry, Faculty of Science and Technology, Thammasat University, Khlong Luang, Pathum Thani 12121, Thailand

**Keywords:** crystal structure, coordination polymers, di­oxy­benzo­quinone, erbium(III), lanthanide

## Abstract

The title lanthanide complex, [Er_2_(C_6_H_2_O_4_)_3_(H_2_O)_6_]·18H_2_O, is isostructural with its La, Gd, Yb and Lu analogues. The Er^3+^ ion, located on a threefold rotation axis, is nine-coordinated in a distorted tricapped trigonal–prismatic geometry, which is completed by six oxygen atoms from three dhbq^2−^ ligands and three oxygen atoms from coordinated water mol­ecules. Each dhbq^2−^ ligand acts in a μ_2_-bis­(bidentate) bridging mode to connect two Er^3+^ ions to form honeycomb (6,3) two-dimensional sheets extending in the *ab* plane, having an Er⋯Er separation of 8.7261 (2) Å. In the crystal, extensive O—H⋯O hydrogen-bonding inter­actions involving the coordinated water mol­ecules and the water mol­ecules of crystallization, as well as the oxygen atoms of the dhbq^2−^ ligands, generate an overall three-dimensional supra­molecular network.

## Abstract 2

The title lanthanide complex is isostructural with its La, Gd, Yb and Lu analogues. The Er^3+^ ion, located on a threefold rotation axis, is nine-coordinated in a distorted tricapped trigonal–prismatic geometry, which is completed by six oxygen atoms from three dhbq^2−^ ligands and three oxygen atoms from coordinated water mol­ecules. Each dhbq^2−^ ligand acts as a μ_2_-bis­(bidentate) bridging mode to connect two Er^3+^ ions to form honeycomb (6,3) two-dimensional sheets extending in the *ab* plane.

## Chemical context   

Over the past few decades, lanthanide-based coordination polymers (LnCPs) have attracted significant attention because their high photoluminescence efficiency and long luminescence lifetime in lighting and full-colour displays (Parker, 2000 ▸; Bünzli & Piguet, 2005 ▸; Cui *et al.*, 2018 ▸). Besides transition metal ions, lanthanide ions feature high coordination numbers and flexible coordination geometries, which facilitate the formation of diverse extended structures. Since lanthanide(III) ions have a high affinity to hard donor atoms, ligands containing oxygen atoms such as carb­oxy­lic acids (Xu *et al.*, 2016 ▸), phospho­ric acids (Mao, 2007 ▸), calixarenes (Ovsyannikov *et al.*, 2017 ▸) and *β*-diketones (Vigato *et al.*, 2009 ▸) have been used extensively in the synthesis of new types of LnCPs. On the basis of the above considerations, we selected 2,5-dihy­droxy-1,4-benzo­quinone (H_2_dhbq) as the ligand to react with erbium(III) nitrate hexa­hydrate under solvothermal conditions to construct a new erbium(III)-based CP, [Er_2_(dhbq)_3_(H_2_O)_6_]·18H_2_O, (I), which is isotypic with its La, Gd, Yb and Lu analogues (Abrahams *et al.*, 2002 ▸). Herein, we report its structure.

## Structural commentary   

**Figure 1:**
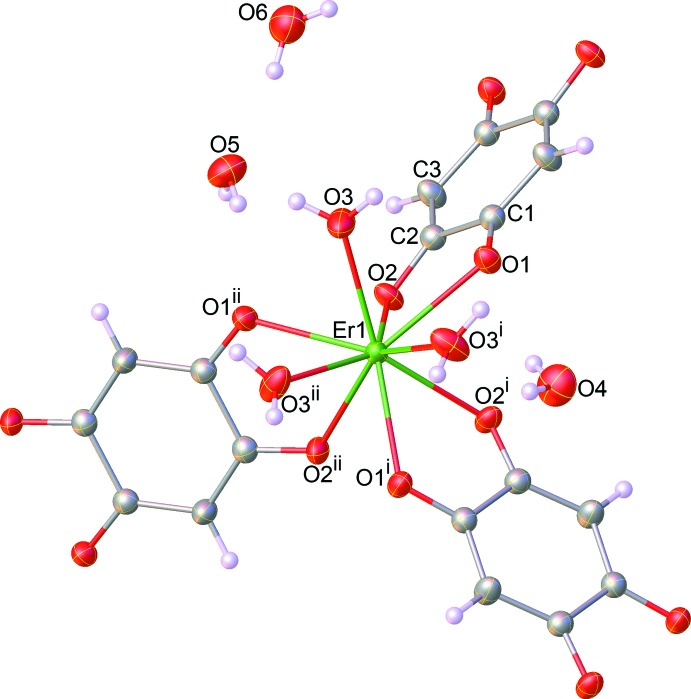
The mol­ecular structure of the title complex, showing selected atom labels. Displacement ellipsoids are drawn at the 50% probability level. Symmetry codes: (i) 1 + *y* − *x*, 1 − *x*, *z*; (ii) 1 − *y*, *x* − *y*, *z*.

**Figure 2:**
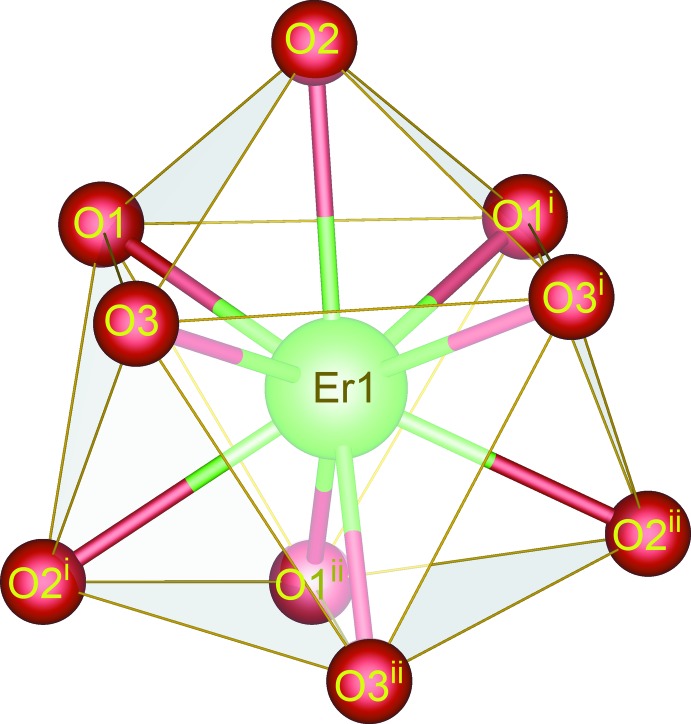
View of the distorted tricapped trigonal–prismatic geometry of the central Er^III^ ion in the title complex. Symmetry codes: (i) 1 + y − x, 1 − x, z; (ii) 1 − y, x − y, z.

**Figure 3:**
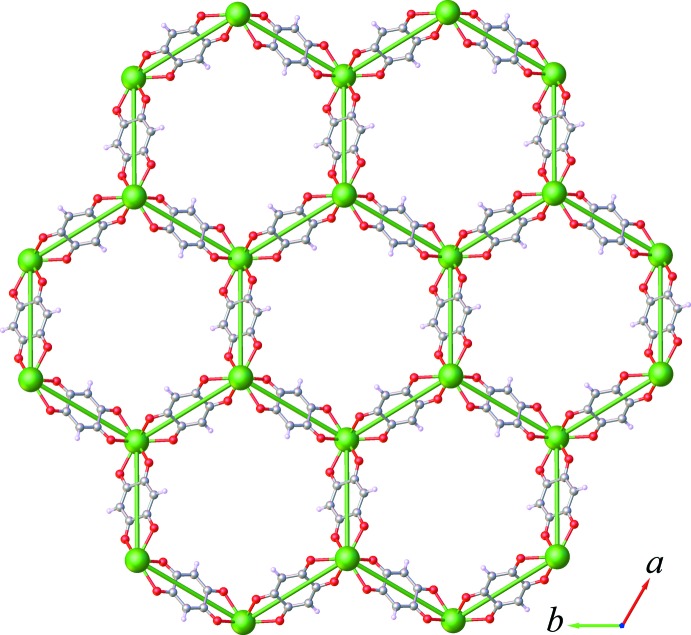
View of the honeycomb (6,3) sheet extending normal to the *c-*axis direction.

The asymmetric unit of (I) contains one third of an Er^3+^ ion, half of a dhbq^2−^ ligand, one coordinated water mol­ecule and three water mol­ecules of crystallization. The Er^3+^ ion is located on a threefold rotation axis, whereas the complete dhbq^2−^ anion is generated by a crystallographic inversion center. As can be seen from Fig. 1 ▸, the Er^3+^ ion is nine-coordinated by six oxygen atoms from three different dhbq^2−^ ligands and three other oxygen atoms from three coordinated water mol­ecules. The coordination polyhedron of the central Er^3+^ ion can best be described as having a distorted tricapped trigonal–prismatic geometry, as depicted in Fig. 2 ▸, in which the O—Er—O bond angles range from 65.01 (5) to 139.97 (7)°. The Er—O bond lengths in the title complex lie between 2.3577 (15) and 2.4567 (15) Å, mean 2.393 Å. The whole dhbq^2−^ anion is nearly planar: the r.m.s. deviation from the mean plane through all of the non-H atoms is 0.021 Å, with a maximum displacement from this plane of 0.033 (2) Å for atom C2. As can be seen from Fig. 3 ▸, the dhbq^2−^ ligand acts in a μ_2_-bis(bidentate) bridging mode, connecting two Er^3+^ ions to form a honeycomb (6,3) sheet extending in the *ab* plane, having a Er⋯Er separation of 8.7261 (2) Å.
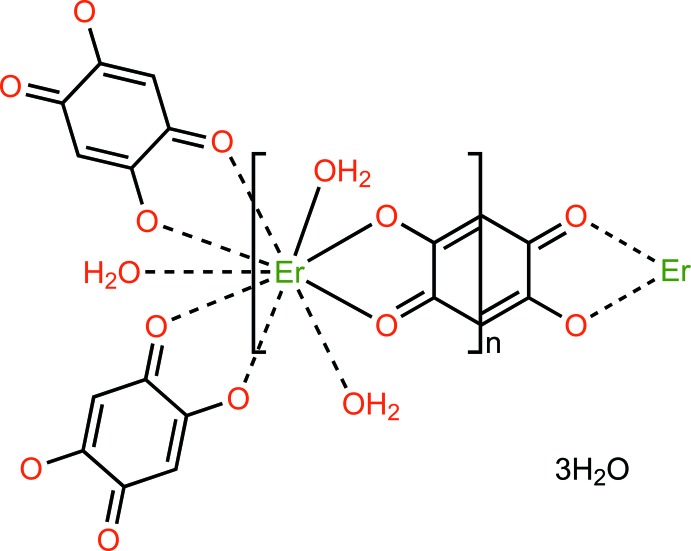


## Supra­molecular features   

**Figure 4:**
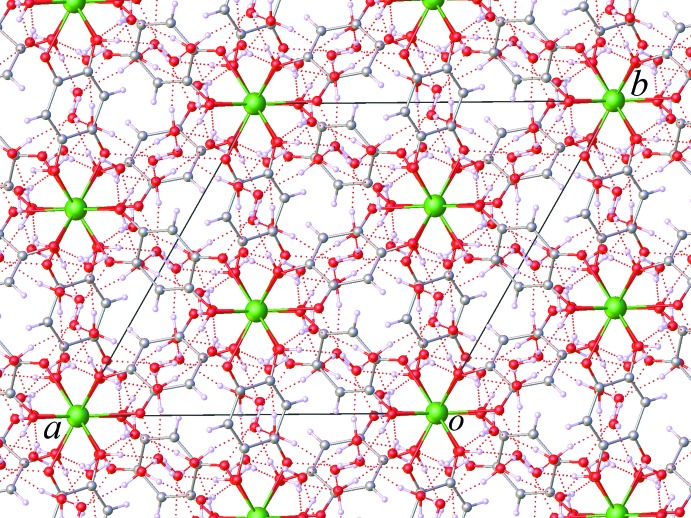
View of the packing in the unit cell of the title complex along the *c* axis. Hydrogen-bonding inter­actions are shown as dashed lines.

In the crystal, extensive O—H⋯O hydrogen-bonding inter­actions (Table 1 ▸) are observed between the oxygen atoms of the coordinated (O3) and lattice (O4 and O5) water mol­ecules as well as between the water (O5 and O6) mol­ecules of crystallization. Other O—H⋯O hydrogen-bonding inter­actions involve O6 and the dhbq^2−^ oxygen atom, and this inter­action further links neighbouring sheets into a three-dimensional supra­molecular structure (Fig. 4 ▸).

## Database survey   

A search of the Cambridge Structural Database (Version 5.39 update February 2018; Groom *et al.*, 2016 ▸) for complexes of dhbq^2−^ ligand gave 94 hits. They include the isotypic crystal structures (Abrahams *et al.*, 2002 ▸) with La (MIZXAU), Gd (MIZXEY), Yb (MIZXIC) and Lu (MIZXOI). In most cases, the dhbq^2−^ ligand acts in a μ_2_-bis­(bidentate) bridging mode to the central metal ions. Comparing the mean *Ln*—O bond length and the unit-cell volume for the title complex with the La, Gd, Yb and Lu analogues (Abrahams *et al.*, 2002 ▸), the values decrease as the ionic radius of the *Ln* ^3+^ ions decreases in the order La [La—O = 2.540 Å, *V* = 3289.3 (16) Å^3^] > Gd [Gd—O = 2.438 Å, *V* = 3162.7 (7) Å^3^] > Er [Er—O = 2.393 Å, *V* = 3107.18 (13) Å^3^] > Yb [Yb—O = 2.377 Å, *V* = 3087.1 (4) Å^3^] > Lu [Lu—O = 2.368 Å, *V* = 3074.2 (4) Å^3^], which is consistent with the lanthanide contraction effect.

## Synthesis and crystallization   

A mixture of Er(NO_3_)_3_·6H_2_O (46.2 mg, 0.1 mmol) and H_2_dhbq (14.2 mg, 0.1 mmol) in distilled H_2_O (4 ml) and DMF (1 ml) was placed in a 20 ml vial and stirred at room temperature for 10 min. The mixture was sealed tightly, placed in an oven and then heated to 358 K under autogenous pressure for 12 h. After the reactor was cooled to room temperature, block-shaped dark-red crystals were filtered off, washed with deionized H_2_O and dried in air at room temperature. Yield: 57% based on Er^III^ source.

## Refinement   

Crystal data, data collection and structure refinement details are summarized in Table 2 ▸. The carbon-bound H atoms were placed in geometrically calculated positions and refined as riding with C—H = 0.93 Å and *U*_iso_(H) = 1.2*U*_eq_(C). The hydrogen atoms of the water mol­ecules were located from difference-Fourier maps but were refined with distance restraints of O—H = 0.84 Å and *U*_iso_(H) = 1.5*U*_eq_(O).

## Supplementary Material

Crystal structure: contains datablock(s) I. DOI: 10.1107/S2056989018017516/hb7788sup1.cif

Structure factors: contains datablock(s) I. DOI: 10.1107/S2056989018017516/hb7788Isup2.hkl

Supporting information file. DOI: 10.1107/S2056989018017516/hb7788Isup3.cdx

CCDC reference: 1884389

Additional supporting information:  crystallographic information; 3D view; checkCIF report

## Figures and Tables

**Table 1:** Hydrogen-bond geometry (Å, °)

*D*—H⋯*A*	*D*—H	H⋯*A*	*D*⋯*A*	*D*—H⋯*A*
O3—H3*A*⋯O6^i^	0.83 (1)	1.94 (1)	2.769 (3)	174 (3)
O3—H3*B*⋯O5^ii^	0.84 (1)	1.94 (1)	2.758 (3)	165 (3)
O4—H4*A*⋯O2^iii^	0.84 (1)	1.92 (1)	2.738 (3)	167 (4)
O4—H4*B*⋯O4^iv^	0.84 (1)	1.98 (1)	2.803 (3)	164 (4)
O5—H5*A*⋯O1^v^	0.85 (1)	2.09 (3)	2.870 (3)	153 (5)
O5—H5*B*⋯O6^vi^	0.84 (1)	1.95 (1)	2.794 (3)	174 (5)
O6—H6*A*⋯O4^vii^	0.85 (1)	1.88 (1)	2.725 (3)	174 (4)
O6—H6*B*⋯O5	0.84 (1)	1.91 (1)	2.747 (3)	169 (5)

Symmetry codes: (i) 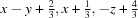; (ii) 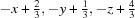; (iii) 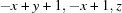; (iv) 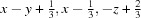; (v) 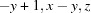; (vi) 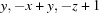; (vii) 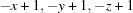.

**Table 2:** Experimental details

Crystal data	
Chemical formula	[Er_2_(C_6_H_2_O_4_)_3_(H_2_O)_6_]·18H_2_O
*M*_r_	1181.13
Crystal system, space group	Trigonal, *R* 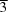
Temperature (K)	296
*a*, *c* (Å)	14.0947 (3), 18.0603 (5)
*V* (Å^3^)	3107.18 (13)
*Z*	3
Radiation type	Mo *K*α
μ (mm^−1^)	4.13
Crystal size (mm)	0.28 × 0.22 × 0.2

Data collection	
Diffractometer	Bruker D8 QUEST CMOS
Absorption correction	Multi-scan (*SADABS*; Bruker, 2016 ▸)
*T*_min_, *T*_max_	0.677, 0.746
No. of measured, independent and observed [*I* > 2σ(*I*)] reflections	32360, 2522, 2108
*R*_int_	0.065
(sin θ/λ)_max_ (Å^−1^)	0.758

Refinement	
*R*[*F* ^2^ > 2σ(*F* ^2^)], *wR*(*F* ^2^), *S*	0.023, 0.042, 1.08
No. of reflections	2522
No. of parameters	118
No. of restraints	8
H-atom treatment	H atoms treated by a mixture of independent and constrained refinement
Δρ_max_, Δρ_min_ (e Å^−3^)	1.67, −1.39

Computer programs: *APEX3* and *SAINT* (Bruker, 2016 ▸), *SHELXT* (Sheldrick, 2015*a* ▸), *SHELXL* (Sheldrick, 2015*b* ▸) and *OLEX2* (Dolomanov *et al.*, 2009 ▸).

## supplementary crystallographic information

### Crystal data

**Table:**

[Er_2_(C_6_H_2_O_4_)_3_(H_2_O)_6_]·18H_2_O	*D*_x_ = 1.894 Mg m^−^^3^
*M**_r_* = 1181.13	Mo *K*α radiation, λ = 0.71073 Å
Trigonal, *R*3	Cell parameters from 8655 reflections
*a* = 14.0947 (3) Å	θ = 2.8–31.7°
*c* = 18.0603 (5) Å	µ = 4.13 mm^−^^1^
*V* = 3107.18 (13) Å^3^	*T* = 296 K
*Z* = 3	Hexagonal prism, dark red
*F*(000) = 1758	0.28 × 0.22 × 0.2 mm

### Data collection

**Table:**

Bruker D8 QUEST CMOS diffractometer	2522 independent reflections
Radiation source: microfocus sealed x-ray tube, Incoatec Iµus	2108 reflections with *I* > 2σ(*I*)
GraphiteDouble Bounce Multilayer Mirror monochromator	*R*_int_ = 0.065
Detector resolution: 10.5 pixels mm^-1^	θ_max_ = 32.6°, θ_min_ = 2.8°
φ and ω scans	*h* = −21→21
Absorption correction: multi-scan (SADABS; Bruker, 2016)	*k* = −19→21
*T*_min_ = 0.677, *T*_max_ = 0.746	*l* = −27→27
32360 measured reflections	

### Refinement

**Table:**

Refinement on *F*^2^	Secondary atom site location: difference Fourier map
Least-squares matrix: full	Hydrogen site location: inferred from neighbouring sites
*R*[*F*^2^ > 2σ(*F*^2^)] = 0.023	H atoms treated by a mixture of independent and constrained refinement
*wR*(*F*^2^) = 0.042	*w* = 1/[σ^2^(*F*_o_^2^) + (0.0042*P*)^2^ + 10.2637*P*] where *P* = (*F*_o_^2^ + 2*F*_c_^2^)/3
*S* = 1.08	(Δ/σ)_max_ = 0.001
2522 reflections	Δρ_max_ = 1.67 e Å^−^^3^
118 parameters	Δρ_min_ = −1.39 e Å^−^^3^
8 restraints	Extinction correction: SHELXL (Sheldrick, 2015b), Fc^*^=kFc[1+0.001xFc^2^λ^3^/sin(2θ)]^-1/4^
Primary atom site location: dual	Extinction coefficient: 0.00020 (2)

### Special details

**Table:**

Geometry. All esds (except the esd in the dihedral angle between two l.s. planes) are estimated using the full covariance matrix. The cell esds are taken into account individually in the estimation of esds in distances, angles and torsion angles; correlations between esds in cell parameters are only used when they are defined by crystal symmetry. An approximate (isotropic) treatment of cell esds is used for estimating esds involving l.s. planes.
Refinement. Refinement of F^2^ against ALL reflections. The weighted R-factor wR and goodness of fit S are based on F^2^, conventional R-factors R are based on F, with F set to zero for negative F^2^. The threshold expression of F^2^ > 2sigma(F^2^) is used only for calculating R-factors(gt) etc. and is not relevant to the choice of reflections for refinement. R-factors based on F^2^ are statistically about twice as large as those based on F, and R- factors based on ALL data will be even larger.

### Fractional atomic coordinates and isotropic or equivalent isotropic displacement parameters (Å^2^)

**Table:**

	*x*	*y*	*z*	*U*_iso_*/*U*_eq_	
Er1	0.6667	0.3333	0.587213 (10)	0.01773 (5)	
O1	0.65928 (12)	0.50374 (13)	0.58327 (9)	0.0236 (3)	
O2	0.54678 (13)	0.33698 (13)	0.49769 (9)	0.0247 (3)	
O3	0.54011 (16)	0.33067 (15)	0.67424 (11)	0.0355 (4)	
H3A	0.557 (3)	0.3878 (16)	0.6975 (16)	0.055 (10)*	
H3B	0.4856 (17)	0.2781 (17)	0.6941 (15)	0.046 (9)*	
O4	0.7266 (2)	0.52214 (19)	0.37540 (12)	0.0455 (5)	
H4A	0.736 (3)	0.494 (3)	0.4136 (13)	0.075 (13)*	
H4B	0.6643 (16)	0.489 (3)	0.356 (2)	0.079 (14)*	
O5	0.29245 (18)	0.15371 (19)	0.57009 (14)	0.0452 (5)	
H5A	0.354 (2)	0.167 (5)	0.587 (3)	0.15 (2)*	
H5B	0.285 (4)	0.138 (4)	0.5247 (8)	0.103 (17)*	
O6	0.18028 (19)	0.2663 (2)	0.57759 (14)	0.0470 (5)	
H6A	0.213 (3)	0.3315 (15)	0.594 (2)	0.091 (16)*	
H6B	0.222 (3)	0.240 (4)	0.576 (2)	0.104 (18)*	
C1	0.58618 (18)	0.50816 (18)	0.54547 (12)	0.0212 (4)	
C2	0.51755 (18)	0.40906 (18)	0.49646 (12)	0.0216 (4)	
C3	0.43354 (19)	0.40401 (19)	0.45415 (13)	0.0256 (5)	
H3	0.3902	0.3424	0.4255	0.031*	

### Atomic displacement parameters (Å^2^)

**Table:**

	*U*^11^	*U*^22^	*U*^33^	*U*^12^	*U*^13^	*U*^23^
Er1	0.01570 (6)	0.01570 (6)	0.02179 (9)	0.00785 (3)	0.000	0.000
O1	0.0218 (8)	0.0214 (8)	0.0293 (8)	0.0121 (7)	−0.0056 (6)	−0.0019 (6)
O2	0.0270 (8)	0.0222 (8)	0.0309 (8)	0.0168 (7)	−0.0069 (7)	−0.0050 (6)
O3	0.0371 (11)	0.0239 (9)	0.0394 (11)	0.0106 (8)	0.0163 (8)	−0.0025 (8)
O4	0.0474 (13)	0.0517 (13)	0.0376 (12)	0.0249 (11)	−0.0043 (10)	0.0091 (10)
O5	0.0335 (11)	0.0505 (13)	0.0505 (14)	0.0202 (10)	0.0094 (10)	0.0035 (11)
O6	0.0434 (13)	0.0436 (14)	0.0534 (14)	0.0212 (11)	0.0057 (10)	0.0082 (11)
C1	0.0202 (10)	0.0208 (10)	0.0221 (10)	0.0099 (9)	0.0003 (8)	0.0004 (8)
C2	0.0215 (10)	0.0217 (10)	0.0226 (10)	0.0115 (9)	0.0011 (8)	0.0015 (8)
C3	0.0259 (11)	0.0216 (11)	0.0318 (12)	0.0137 (10)	−0.0077 (9)	−0.0069 (9)

### Geometric parameters (Å, º)

**Table:**

Er1—O1^i^	2.4567 (15)	O3—H3B	0.836 (10)
Er1—O1	2.4567 (15)	O4—H4A	0.838 (10)
Er1—O1^ii^	2.4567 (15)	O4—H4B	0.842 (10)
Er1—O2^ii^	2.3578 (15)	O5—H5A	0.845 (10)
Er1—O2^i^	2.3577 (15)	O5—H5B	0.844 (10)
Er1—O2	2.3578 (15)	O6—H6A	0.848 (10)
Er1—O3	2.3636 (18)	O6—H6B	0.843 (10)
Er1—O3^i^	2.3636 (18)	C1—C2	1.523 (3)
Er1—O3^ii^	2.3637 (18)	C1—C3^iii^	1.398 (3)
O1—C1	1.263 (3)	C2—C3	1.381 (3)
O2—C2	1.273 (3)	C3—C1^iii^	1.398 (3)
O3—H3A	0.831 (10)	C3—H3	0.9300
			
O1^i^—Er1—O1	119.917 (4)	O3—Er1—O1^ii^	139.97 (6)
O1^ii^—Er1—O1^i^	119.917 (4)	O3—Er1—O1	68.54 (6)
O1^ii^—Er1—O1	119.916 (4)	O3^ii^—Er1—O1^ii^	68.54 (6)
O2^i^—Er1—O1^i^	65.01 (5)	O3^i^—Er1—O1^ii^	70.00 (6)
O2^ii^—Er1—O1	69.91 (5)	O3^i^—Er1—O1^i^	68.54 (6)
O2^ii^—Er1—O1^ii^	65.01 (5)	O3—Er1—O1^i^	70.00 (6)
O2—Er1—O1	65.01 (5)	O3^i^—Er1—O3^ii^	80.60 (8)
O2—Er1—O1^ii^	134.93 (5)	O3^i^—Er1—O3	80.60 (8)
O2^i^—Er1—O1^ii^	69.91 (5)	O3—Er1—O3^ii^	80.60 (8)
O2^ii^—Er1—O1^i^	134.93 (5)	C1—O1—Er1	119.88 (14)
O2—Er1—O1^i^	69.91 (5)	C2—O2—Er1	123.42 (14)
O2^i^—Er1—O1	134.93 (5)	Er1—O3—H3A	118 (2)
O2^i^—Er1—O2^ii^	78.15 (6)	Er1—O3—H3B	131 (2)
O2^i^—Er1—O2	78.15 (6)	H3A—O3—H3B	109 (3)
O2^ii^—Er1—O2	78.15 (6)	H4A—O4—H4B	117 (4)
O2^ii^—Er1—O3^ii^	85.01 (7)	H5A—O5—H5B	112 (5)
O2—Er1—O3^ii^	134.96 (6)	H6A—O6—H6B	112 (4)
O2^i^—Er1—O3^ii^	138.45 (6)	O1—C1—C2	115.38 (19)
O2^i^—Er1—O3	134.96 (6)	O1—C1—C3^iii^	124.7 (2)
O2—Er1—O3	85.01 (7)	C3^iii^—C1—C2	119.92 (19)
O2^ii^—Er1—O3	138.45 (6)	O2—C2—C1	114.28 (19)
O2^i^—Er1—O3^i^	85.01 (7)	O2—C2—C3	125.4 (2)
O2^ii^—Er1—O3^i^	134.96 (6)	C3—C2—C1	120.26 (19)
O2—Er1—O3^i^	138.46 (6)	C1^iii^—C3—H3	120.1
O3^ii^—Er1—O1^i^	139.97 (7)	C2—C3—C1^iii^	119.8 (2)
O3^i^—Er1—O1	139.97 (6)	C2—C3—H3	120.1
O3^ii^—Er1—O1	70.00 (6)		
			
Er1—O1—C1—C2	−8.1 (2)	O2^ii^—Er1—O1—C1	96.59 (16)
Er1—O1—C1—C3^iii^	172.37 (18)	O2^ii^—Er1—O2—C2	−86.3 (2)
Er1—O2—C2—C1	13.9 (3)	O2^i^—Er1—O2—C2	−166.47 (17)
Er1—O2—C2—C3	−167.71 (18)	O2—C2—C3—C1^iii^	−176.2 (2)
O1^ii^—Er1—O1—C1	139.76 (13)	O3^ii^—Er1—O1—C1	−171.43 (17)
O1^i^—Er1—O1—C1	−34.5 (2)	O3^i^—Er1—O1—C1	−126.15 (16)
O1^ii^—Er1—O2—C2	−121.30 (16)	O3—Er1—O1—C1	−83.94 (16)
O1—Er1—O2—C2	−13.10 (16)	O3^ii^—Er1—O2—C2	−15.9 (2)
O1^i^—Er1—O2—C2	126.04 (18)	O3—Er1—O2—C2	55.53 (17)
O1—C1—C2—O2	−3.1 (3)	O3^i^—Er1—O2—C2	125.30 (17)
O1—C1—C2—C3	178.4 (2)	C1—C2—C3—C1^iii^	2.1 (4)
O2^i^—Er1—O1—C1	48.94 (18)	C3^iii^—C1—C2—O2	176.4 (2)
O2—Er1—O1—C1	10.65 (15)	C3^iii^—C1—C2—C3	−2.1 (4)

Symmetry codes: (i) −*y*+1, *x*−*y*, *z*; (ii) −*x*+*y*+1, −*x*+1, *z*; (iii) −*x*+1, −*y*+1, −*z*+1.

### Hydrogen-bond geometry (Å, º)

**Table:**

*D*—H···*A*	*D*—H	H···*A*	*D*···*A*	*D*—H···*A*
O3—H3*A*···O6^iv^	0.83 (1)	1.94 (1)	2.769 (3)	174 (3)
O3—H3*B*···O5^v^	0.84 (1)	1.94 (1)	2.758 (3)	165 (3)
O4—H4*A*···O2^ii^	0.84 (1)	1.92 (1)	2.738 (3)	167 (4)
O4—H4*B*···O4^vi^	0.84 (1)	1.98 (1)	2.803 (3)	164 (4)
O5—H5*A*···O1^i^	0.85 (1)	2.09 (3)	2.870 (3)	153 (5)
O5—H5*B*···O6^vii^	0.84 (1)	1.95 (1)	2.794 (3)	174 (5)
O6—H6*A*···O4^iii^	0.85 (1)	1.88 (1)	2.725 (3)	174 (4)
O6—H6*B*···O5	0.84 (1)	1.91 (1)	2.747 (3)	169 (5)

Symmetry codes: (i) −*y*+1, *x*−*y*, *z*; (ii) −*x*+*y*+1, −*x*+1, *z*; (iii) −*x*+1, −*y*+1, −*z*+1; (iv) *x*−*y*+2/3, *x*+1/3, −*z*+4/3; (v) −*x*+2/3, −*y*+1/3, −*z*+4/3; (vi) *x*−*y*+1/3, *x*−1/3, −*z*+2/3; (vii) *y*, −*x*+*y*, −*z*+1.
